# Aerial Continuum Manipulation: A New Platform for Compliant Aerial Manipulation

**DOI:** 10.3389/frobt.2022.903877

**Published:** 2022-08-05

**Authors:** Amir Jalali, Farrokh Janabi-Sharifi

**Affiliations:** Robotics, Mechatronics, and Automation Laboratory, Department of Mechanical and Industrial Engineering, Ryerson University, Toronto, ON, Canada

**Keywords:** aerial manipulation, continuum robots, compliance, cooperative, conceptual design

## Abstract

Traditional aerial manipulation systems were usually composed of rigid-link manipulators attached to an aerial platform, arising several rigidity-related issues such as difficulties of reach, compliant motion, adaptability to object’s shape and pose uncertainties, and safety of human-manipulator interactions, especially in unstructured and confined environments. To address these issues, partially compliant manipulators, composed of rigid links and compliant/flexible joints, were proposed; however, they still suffer from insufficient dexterity and maneuverability. In this article, a new set of compliant aerial manipulators is suggested. For this purpose, the concept of aerial continuum manipulation system (ACMS) is introduced, several conceptual configurations are proposed, and the functionalities of ACMSs for different applications are discussed. Then, the performances of proposed aerial manipulators are compared with conventional aerial manipulators by implementing available benchmarks in the literature. To enhance the comparison, new features with related benchmarks are presented and used for evaluation purposes. In this study, the advantages of ACMSs over their rigid-link counterparts are illustrated and the potential applications of ACMSs are suggested. The open problems such as those related to dynamic coupling and control of ACMSs are also highlighted.

## Introduction

Owing to the advantages of UAVs, there has been a recent shift of focus to expanding their use from passively “seeing” the environment to physical (active) interactions with their surroundings, leading to the introduction of aerial manipulation systems (AMSs) (Khamseh et al., 2018; Ruggiero et al., 2018). Aerial manipulators usually consist of a traditional flying platform (UAVs) equipped with various manipulation mechanism(s) (Khamseh et al., 2018). Dexterous robotic arms constitute the most conventional manipulators which are attached directly to the UAVs, mostly underneath. The arm provides manipulation and reach capabilities, particularly for accessing hard-to-reach spaces in cluttered environments, e.g., for operations such as pick-and-place, load transportation, and force exertion. However, despite evident advantages and potentials of conventional aerial manipulators, there are still challenges that need to be addressed:

### Payload Weight

As degrees of freedoms (DOFs) of aerial manipulators are desired to increase, more rigid arms and actuators were implemented, leading to increasing the weight of conventional manipulators. To resolve this issue, several solutions were considered in the literature. Conventionally, more powerful aerial platforms like helicopters were used (Pounds et al., 2011a; Pounds et al., 2011b; Pounds et al., 2011c). However, using large aerial platforms restricts the aerial manipulators for outdoor applications or for missions with sufficiently clear spaces. Hence, they are not suitable for indoor or unknown environments, where there might be several obstacles with unknown sizes and shapes. As another solution, several tethered aerial robot systems were introduced (Lee D. et al., 2020; Lee S. M. et al., 2020; Chien et al., 2021) to transfer the actuation box on the ground and keeping the original aerial platform as small as possible. Concerning the latter one, the aerial-ground transmission line will produce limitations for using the aerial manipulators in confined environments or for long distance indoor/outdoor missions. Hence, there is a conflict between the payload capacity and DOFs of the current aerial manipulators that needs to be resolved. Furthermore, a viable solution should be in manipulator level, avoiding any limitations for AMS applications.

### Dexterity and Maneuverability

As discussed above, trade-off between DOFs and weight of conventional manipulators restricts their dexterity and maneuverability.

### Compliance

With rigid-link arms, uncertainties related to the shape and pose (position and orientation) of the objects would readily lead to the failure of grasp and aerial manipulation tasks. Reaching challenging and hardly accessible sites also requires significant dexterity that cannot be readily achieved with traditional rigid-link arms as they may require intricate fabrication, integration, and instrumentation (Webster et al., 2008). Hence, compliance were introduced as a desirable feature for aerial manipulators, especially for those which were intended to physically interact with the environment since the stability of the floating base may be compromised by the forces caused by the dynamic coupling with the manipulator. Besides, compliance will promote reach and dexterity of aerial manipulators, particularly in missions with unknown objects in unstructured and cluttered environments (Ruggiero et al., 2018). For this purpose, researchers either suggested mechanical compliance (Wisanuvej et al., 2014; Suarez et al., 2015; Yuksel et al., 2015; Bartelds et al., 2016) or treated the issue at the control level (Antonelli et al., 2016; Suarez et al., 2018; Hamaza et al., 2019). For the former type of solution, researchers substituted rigid-link manipulators with partially compliant ones which are composed of both rigid bodies and flexure joints (Teo et al., 2014). By the latter set of solutions, virtual impedance control considering both rigid-link and partially compliant aerial manipulators have been investigated. However, the issues related to robustness and complexity of the control-based solutions have limited their applications. Therefore, the problem of designing aerial compliant manipulation systems remains open.

To overcome the aforementioned limitations, we have proposed the new paradigm of aerial continuum manipulation in which compliant continuum robots (CRs) are integrated into aerial vehicles. CRs usually involve a continuous backbone (with infinite DOFs) enabling them to adapt their shapes readily to the shapes of the objects during grasp, and maneuver easily in crowded environments. This property also makes them a safer choice to interact with humans for collaborative tasks. Additionally, CRs come with the potential advantage of high dexterity/weight ratio, higher output force/weight ratio, and stiffness modulation (Peng et al., 2021). The aforementioned features would also allow design of new compound continuum robots for manipulation tasks, enabling new set of applications for aerial manipulation.

In summary, despite the desired characteristics of conventional aerial manipulators, they are still premature and accommodating nature-inspired mechanisms/designs could advance the functionality of aerial manipulators. Soft continuum robots offer all necessary features of an anticipated aerial robot for many day-to-day applications.

## Brief Literature Survey on Similar Works

The literature on aerial continuum manipulation has focused on implementing continuum arms in the *gripper* and/or *manipulator* level.

At the gripper level, Mishra et al. (2018) integrated a soft silicone robotic grasper with a hexacopter for damp or wet applications like picking up contaminants or sampling from the water surface. A soft gripper with four soft fingers has also been presented in (Fishman and Carlone, 2021). In their design, four independent tendon-driven continuum robots were attached to the quadrotor’s base to provide a 4-finger continuum gripper. The results proved the advantages of softness in aerial manipulation, e.g., in adaptation to deviations from the nominal quadrotor trajectory and mitigating the contact impact on the quadrotor and the target, thus enabling successful grasps from a wide range of initial conditions in which conventional solutions using rigid-link grippers fail. In summary, with their focus being on grasping the objects, the manipulation capability of gripper-level implementations is limited.

At the manipulator level, several works (Kim et al., 2018; Zhang et al., 2019; Samadikhoshkho et al., 2020; Chien et al., 2021; Peng et al., 2021) have implemented soft/continuum robots for aerial manipulations. In (Kim et al., 2018; Zhang et al., 2019), an origami-folding inspired design and fabrication approach has been proposed for developing semi-soft robotic arms in which materials with inherent compliance were employed. The prototypes provided extensible and foldable arms with pneumatic (Zhang et al., 2019) and electric (Kim et al., 2018) actuation. In (Zhang et al., 2019), two arms were integrated on a micro aerial vehicle (MAV) to obtain a platform with the potential of aerial manipulation capabilities in confined and hard-to-reach areas whereas the platform presented in (Kim et al., 2018) was proposed for conventional drone sizes. Because of the pneumatic actuation and the micro size aerial platform, the payload capacity of the proposed ACM in (Zhang et al., 2019) is too small. Furthermore, as (Zhang et al., 2019) and (Kim et al., 2018) utilized foldable arms, they can be used just for straight line contraction and extension and hence, the AMS needs to be exactly above the object for further manipulation. In our previous work (Samadikhoshkho et al., 2020), a single-section tendon-driven continuum robot was used as the robotic manipulator and a robust adaptive control approach for the position control of ACM was proposed. The motivation for this work was illustrating an efficient control algorithm to resolve the issues related to strong dynamic coupling between the aerial platform and continuum manipulator. Inspired by our previous work (Samadikhoshkho et al., 2020), a tendon-driven aerial manipulator was developed in (Peng et al., 2021) and the experimental results proved the ability of such arms to provide comparable payload capacity with great dexterity. This study tried to validate the concept introduced in (Samadikhoshkho et al., 2020) while modifying the design of the continuum manipulator. In (Samadikhoshkho et al., 2020) and (Peng et al., 2021), only the applied control algorithm and the potential capabilities of single section-continuum robot was evaluated and the passive functionality of the robot, its possible improvements and evaluation measures, and practical applications of ACMSs were not discussed. As another study (Chien et al., 2021), developed a tethered single-section tendon-driven aerial continuum manipulator where the power on-board is transmitted from the ground station. However, because of the ground-aerial platform transmission line, it is capable of performing limited manipulation tasks in a close distance.

In this article, being inspired by elephant trunks or octopus arms, we envision introducing a novel concept of continuously flexible or continuum robots for aerial manipulation. We propose tendon-driven *concentric-tube CRs* as the core element of aerial continuum manipulation systems (ACMSs). Next, various configuration designs for ACMSs are given. The advantages of the proposed ACMSs over their rigid-link counterparts are provided and possible challenges are discussed. The article is dedicated to concept explanation and hence, modeling and control aspects of ACMSs are left to our future research. Finally, benchmarks are provided to compare ACMSs with their counterparts (aerial rigid-link manipulation systems or ARMSs).

## The Proposed Aerial Continuum Manipulator

The envisaged continuum manipulator for aerial missions should be light, fully flexible, and with the possibility of simultaneous bending and elongation for reaching hard-to-access spaces in cluttered environments. Furthermore, the manipulator is expected to perform manipulation tasks ranging from simple grasping to complicated missions. Hence, it needs to modulate its payload capacity and stiffness, on-demand, in response to the surrounding.

CRs present flexible and light manipulators with a higher payload-to-weight ratio than rigid robots. The actuation type is the main parameter of determining the CRs’ payload capacity. Concerning the actuation type, continuum arms can be classified into five groups: tendon-driven, concentric tube, pneumatic, hydraulic, and magnetically actuated CRs. Tendon-driven CRs are slender continuum arms that have a reasonable payload to weight ratio. They present a compact robot structure and provide position accuracy and operation precision in constrained space. Conventional concentric-tube CRs work with pre-curved tubes and generally have a small payload capacity. The main feature of these robots is their modular design, maintaining the compactness and miniaturization of the robot. To combine the compactness of concentric tube CRs with higher payload capacities, tendon-bent concentric tube CRs have been proposed (Amanov et al., 2021; Janabi-Sharifi et al., 2021). Pneumatic CRs are more conventional than hydraulic CRs. The weight of the robot body is light and the structure is simple. They can carry significantly heavy payloads. However, the cross-section of the arm is almost large which limits its access to confined spaces. Hence, it is a generic challenge to construct slender manipulators with hydraulic and pneumatic CRs (Wang et al., 2021). Magnetic CRs are recently introduced to the community and are currently implemented in small-scale manipulators (Kim et al., 2019). The magnetic drive is equipped with a magnetic field generator outside, which will make the robot structure less compact. Furthermore, the robot’s motion range will be limited to the magnetic field working area, which brings great restrictions (Zhong et al., 2020). Hence, they have not been developed for large-scale industrial manipulators yet.

**FIGURE 1:**
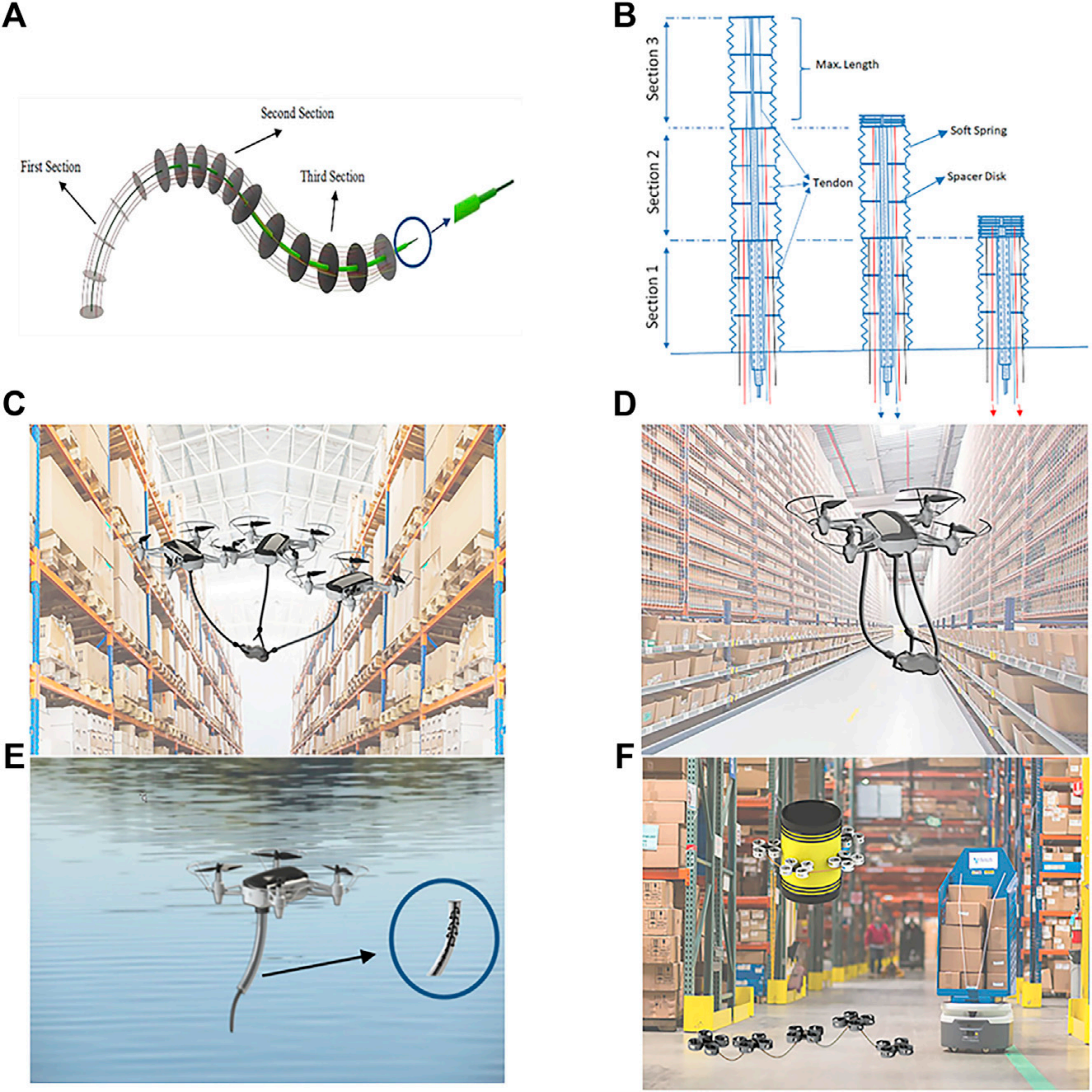
**(A)** Schematic configuration of a 3 section tendon-driven concentric tube CR; **(B)** proposed design for a typical CR with three tubes. Conceptual platforms: **(C)** cooperative platform-type (i); **(D)** cooperative platform-type (ii); **(E)** self-container; and **(F)** whole-body.

In this study *tendon-driven concentric tube CRs* are suggested for aerial manipulation. A schematic configuration of the proposed manipulator has been shown in Figure 1A. The concentric tube CR provides the elongation of the robot while bending. On the other hand, tendon actuation will promote the payload capacity of conventional pre-curved concentric tubes. Furthermore, it decouples the section lengths and curvatures of the robot and provides an accurate path following motions (Amanov et al., 2021). Hence, the proposed tendon-driven concentric tube CR presents miniaturized lightweight manipulators adapted for operation in hardly accessible environments. To address payload modulation, cooperative continuum robots are proposed in which the number of involved arms will be adjusted in different missions based on the targeted payload. Consequently, the stiffness modulation will automatically be accommodated in the aerial manipulator as the operator can modify its overall stiffness by the number of involved arms. Regarding the stiffness modulation, to maintain position accuracy, the manipulator needs to vary the stiffness independently of the end-effector position (Giannaccini et al., 2018) which can easily be achieved by cooperative manipulation. Continuum robots share other advantages such as whole-body manipulation which indicates the ability to use the entire length of the arm instead of relying solely on its end-effector. It advances object manipulations with unknown size and shape and consequently, increases the manipulation success rate.

Regarding *aerial* continuum manipulation, the flexibility of the arm in ACMS also leads to its increased ability for recovering from anomaly conditions such as possible collisions with the environment. The continuum manipulator could easily shrink underneath the UAV when it is not in operation which increases the maneuverability of the flying platform. Furthermore, the continuum manipulators can be used as contact/force sensors during operation which is a great advantage in conducting compliant motions/precise missions where extra force sensor attachment is not feasible. These advantages are almost impossible with rigid-link aerial manipulators. Regarding cooperative arrangement, it also enables implementation of different arms’ types, according to the specified mission, offering great flexibility in design.

## Aerial Continuum Manipulation System: Mathematical Framework

In the following, the general framework for mathematical modeling of an ACMS consists of a tendon-driven concentric tube manipulator attached to the quadrotor has been presented.

### Aerial Manipulation System

Due to the advantages such as lightweight, high mobility, and versatility, quadrotors have usually been purposed for AMSs. Accordingly, the equations of motions for the quadrotor can be describe as follows (Samadikhoshkho et al., 2020; Hashemi et al., 2021):

$${\dot{Y}}_{i}=g_{i}\left(R_{q},v_{q},\omega_{q},r,\tau \right)\;\;\;\;\;\;i=1,2,\ldots ,4$$ (1)

where $Y=\left(p_{q},R_{q},v_{q},\omega_{q}\right)$ in which $p_{q}\in {\mathbb{R}}^{3}$ is the position of the quadrotor, $R_{q}\in \mathrm{SO}\left(3\right)\;$ is the rotation matrix, $v_{q}\in {\mathbb{R}}^{3}\;$ and $\omega_{q}\;\in {\mathbb{R}}^{3}$ are the linear and angular velocities of the quadrotor, $r\in {\mathbb{R}}^{3}\;$ is the thrust vector, and $\tau \;\in {\mathbb{R}}^{3}$ is the control torque.

### Tendon-Driven Concentric Tube CR

Concerning the tendon-driven actuation, elastic backbones with *m* continuous channels for *m* tendons provide the basic idea of tendon-driven CRs. In practice, distributing multiple spacer disks (with holes located in the specified offset from the center of the disk) along the backbone can accurately approximate the backbone with continuous channels. Regarding concentric-tube robots, they theoretically consist of n tubes with initial lengths of $L_{i},\;i=1,\;3,\ldots ,n$ , the inner radius $r_{i}^{i}$ , and the outer radius $r_{i}^{o}$ where ${\left(.\right)}^{i}$ and ${\left(.\right)}^{o}$ indicate the inner and outer radius of the tubes, respectively, and i=1 relates to the innermost tube. It is assumed that $r_{i}^{o}=r_{i+1}^{i}$ , $L_{i-1}>L_{i}$ , and the tubes are initially straight. When it comes to tendon-driven concentric tube CRs, we would have multiple continuum tubes nested to each other where the spacer disks are placed just on the exposed section of each tube. All tubes can translate and are tendon actuated in bending. A schematic configuration of the robot has been shown in Figure 1B. Elongation of the tubes produces difficulties regarding the distribution of the spacer disks. As the exposed portion of the tubes changes, we need a mechanism to automatically spread the spacer disks along the exposed section. For this purpose, magnetic field (Amanov et al., 2021) and spring force (Janabi-Sharifi et al., 2021) have been proposed. Soft springs are placed between each two consecutive spacer disks and, compared to magnetically distributed disks, provide the advantages of low inertia and negligible stiffness variation of the arm, offering a practical solution with small effects on the modeling approaches. Using soft springs, by pulling out a tube from the base, the springs are compressed which allows the robot to reduce its length. Similarly, by inserting each tube in, because of the spring’s forces, the spacer disks will be uniformly distributed along the exposed portion of the tubes, making the robot ready for tendon actuation. In this structural configuration, the tubes never twist relative to others as the tendons of tube i pass through the holes placed on the spacer disks of all previous tubes which restricts the twisting of each tube relative to others. So, in this robot, the only possible movements are the extension of the tubes and their bending.

In this design, as the exposed portion of each tube should be uniformly covered by the spacer disks, the number of disks and the exposed length of each section should be predetermined in the design phase. This will produce a length limitation for the whole robot. For example, for the concentric tube robot consisting of 3 tubes shown in Figures 1B, if the predefined exposed portion of tubes are $l_{i},\;i=1,\;2,\;3$ , the maximum length of the robot would be $l_{1}+l_{2}+l_{3}$ . If the length exceeds this criterion there is at least one section with inconsistent disk distribution.

Based on the above hypothetical design, considering the small stiffness of the springs, neglecting the friction force and looseness between the tubes (Rucker et al., 2010), the equations of motion of each section based on the Cosserat rod model serve as the following strong differential equations form:

$$\partial_{s}\mathrm{n+f=}\rho AR\left(\hat{\omega }q+\partial_{t}q\right),\;\partial_{s}\mathrm{m+l=}\partial_{t}\left(\rho \mathrm{RJ\omega}\right)-\partial_{s}\left(\hat{p}\right)n.$$ (2)

The variables n(s,t) $\in {\mathbb{R}}^{3}\;$ and m(s,t) $\in {\mathbb{R}}^{3}\;$ are the internal force and moment, respectively, while $f\in {\mathbb{R}}^{3}$ and $l\in {\mathbb{R}}^{3}$ are externally applied forces and moments, respectively, R(s,t)∈SO(3) is the material orientation, $p\left(s,t\right)\in {\mathbb{R}}^{3}$ is the centerline curve, all in the global coordinate frame. The external loads and moments f and l cover a variety of loads from moving effects of the aerial platform (Samadikhoshkho et al., 2020) to tendon tensions (Janabi-Sharifi et al., 2021), robot weight, and air damping. q(s,t) and ω(s,t) are the linear and angular velocities in the local coordinate frame, respectively, while $\partial_{s}$ and $\partial_{t}$ indicate the derivation with respect to *s* and *t*, respectively. Also, ρ , A , and J denote the density, the cross-section of the arm, and the rotational inertia matrix, respectively, and $\hat{.}$ maps SO(3) to so(3) . Furthermore, n and m in Eq. 1 are related to the kinematic variables through a suitable material constitutive law. It should be noted that as the number of tubes in each section differs, the geometrical properties of the cross-section, as well as the tendon offsets from the backbone change accordingly.

Regarding the length change of the tubes, as the insertion speed and acceleration occur at much smaller frequencies than the natural frequencies of the continuum robot, it is practically reasonable to assume that the insertion speed and acceleration of the tubes are relatively small, making it possible to quasi-statically model the insertion of the tube with the extension rate $q_{ins}$ (Janabi-Sharifi et al., 2021). Concerning quasi-static insertion, there are several options such as element generation algorithms that accurately and precisely model the insertion with no effects on the rest of the solution approach (in this case, tendon-based bending actuation solution).

To sum up, the modeling method considers the insertion of the tubes as well as their bending. For this purpose, the length of the tubes will be updated based on the given quasi-static insertion rate and then the tendon tensions will bend the whole robot based on Eq. 2.

Aside from the equations of motion, the Boundary Conditions (BCs) have to be determined. Besides, there are other consistency conditions, maintaining the connection of quadrotor-manipulator, that needs to be satisfied during the solution. Regarding the boundary conditions, the tendon tensions produce concentrated loads and moments $F\in {\mathbb{R}}^{3}$ and $L\in {\mathbb{R}}^{3}$ at the distal end. Hence, if *L* is the total length of the CR, we would have:

$$s=L\to \{\begin{array}{c} n=F\\ m=L \end{array}.$$ (3)

When it comes to the consistency conditions, the following relations need to be maintained at the proximal end of the CR, where the robot attached to the quadrotor at point A:

$$s=0\to \{\begin{array}{c} p=p_{q}\left(A\right)\\ R=R_{q}\left(A\right)\\ q=v_{q}\left(A\right)\\ \omega =\omega_{q}\left(A\right) \end{array}.$$ (4)

## Aerial Continuum Manipulation Platforms

In the following, we tailored the introduced novel concepts of ACMS for several applications. The proposed aerial platforms are expected to either simplify the mission or improve the manipulation performance.

### Cooperative Aerial Manipulation

A cooperative manipulation can be achieved by 1) multiple drones each using its own CR (Figure 1B) or 2) one drone armed with multiple CRs (Figure 1C). The first arrangement (Figure 1B) leads to increased workspace and fault tolerance pertaining to the UAV failures. For instance, it enables the robots to reach an object surrounded by the obstacles from different paths. Due to the increased space requirements for safe maneuver of separate UAVs, this assembly is not recommended for operation in small flight space. Alternatively, the second arrangement (Figure 1C) provides a compact solution for narrow space missions with cost of possibly lower payload capacity compared to the first arrangement due to the involvement of only a single UAV. Both arrangements suggest a good substitute for cable-suspended transportation where additional manipulation force-torque exertion beyond tensioning the cables might also be needed.

### Continuum Arms as Containers

This ACMS configuration (Figure 1D) will provide the advantage of sampling from the environment, specifically liquid samples, and storing samples while performing other manipulations. In this application, the innermost tube of the concentric-tube CR is the suction head and the other tubes are used as containers, providing a self-container manipulator. With multi-link rigid robots, the above trait will not be achieved unless using different parts as the manipulator and container, leading to larger footprint and potentially limited manipulation workspace. In the proposed design (Figure 1D), the outermost tube is a hollow cylinder that is tightly sealed at the tip to prevent leakage.

Compared to a separate container, a self-container CR has reduced effects on the center-of-gravity (CoG) of the AMS, leading to a smaller form factor for aerial manipulation to maintain dynamic stability and handling. These advantages will accompany intrinsic characteristics of CRs, e.g., forming flexible postures during sampling which makes the sampling procedure easier. The benefits of this feature will be more highlighted when the self-container arm is used in a cooperative aerial manipulation arrangement, converting the ACMS to a flying lab for aerial rapid tests.

### Whole-Body Aerial Manipulation

Object transportation is one of the well-known applications of aerial manipulators. In this context, both robotic manipulators and single grippers have been used. With robotic manipulators of several DOFs, the stabilization of the orientation for the whole system becomes problematic. Regarding single grippers, an offset between the CoG of the aerial robot and the object is expectable which also leads to flight instability. The bigger the object is, the worse this issue persists. Another challenge will appear when the AMS is expected to grasp large objects, bigger than the gripper. For this purpose, (Zhao et al., 2017) proposed an aerial manipulation in which the whole body of the aerial robot will grasp and lift large objects. Hence, it will guarantee orientation stability. This idea was achieved by introducing transformable multirotor comprising link modules with built-in propellers. The joints have the same rotational axis which provides two-dimensional transformation. This transformable multirotor can be treated as a single gripper to hold an object by the whole body. This structural modification of AMSs will provide the capability of gripping large objects with minimum effect on the CoG of the aerial system. However, the shape of the object should be known for optimal grasping before aerial mission as there is a relation between the number of links and the sides of the objects. This restriction will limit the application of these aerial robots, especially for unknown environments and objects. Furthermore, the rigid-link arms would not easily create distributed contact grasp forces/torques, leading to higher sensitivity of resultant grasps to their deviations from optimal grasping forms. This, in turn, would lead to higher force/torque applications that may exceed the object surface limits (especially in soft objects).

To address this shortcoming, a whole-body continuum aerial manipulation is proposed (Figure 1E) in which CRs replace rigid-link arms to increase the adaptability of the whole-body gripper to the object shape. Also due to the compliant structure of the CRs, this assembly will provide the capability of force sensing along the continuum gripper to maintain the related threshold for maximum applied forces. Furthermore, compared to its conventional counterpart, the proposed manipulation will provide improved stability for the aerial platform due to low-stiffness coupling between two consecutive drones.

## Functionality and Performance Analysis of ACMSs

Practical benchmarks are crucial for evaluating the performance of any AMS including ACMSs. The introduced benchmark is an extension of the previous work (Suarez et al., 2020) to highlight the significant features of the involved arms. The benchmark in (Suarez et al., 2020) was motivated by the convenience to facilitate the evaluation and comparison in the performance of different AMSs in terms of time metrics, positioning accuracy and repeatability, control errors in grasping tasks, or response to physical interactions. In this study, two groups of benchmarks were proposed: evaluation of the manipulator’s performance in 1) the test bench (with arm only) and 2) indoor/outdoor flight test. Regarding these futures, the introduced benchmarks with related metrics have been summarized in Table 1. We have also added other metrics to enhance the evaluation: jammability, agility, and energy performance.

**TABLE 1:** Features and related benchmarks to evaluate the performance of AMS. The last two columns compared the ACMSs with their rigid-link counterparts (∼: capable, ✓: superior).

	Feature	Benchmark	Metrics	Rigid-link manipulator	Continuum manipulator
Test bench	**Trajectory accuracy**	Draw a circle with a pen attached to the end effector and compare it w.r.t. a ground truth	Max error, amplitude, time	N/A	N/A
	**Positioning accuracy and repeatability**	Draw N marks with a pen in two areas separated a certain distance and compared them with the reference points	Maximum error, distance, time	N/A	N/A
	**Payload capacity**	Lift a mass attached at the end effector	Payload mass, torque, PWM signal of the servo	✓	✓
	**Force/load estimation and control**	Apply a sequence of force references in different axes	Amplitude force, max. error, rise time, overshoot	✓	✓
	**Collision detection and reaction**	Hit an object while the manipulator is moving, detect the impact, and react going backward	Manipulator speed, displaced distance	**∼**	✓
	**Agility**	Apply quick direction change during forward motion of the arm	The average manipulation time	✓	**∼**
Aerial test	**Object grasping**	Grab an object located in a tool bench at a given distance and height w.r.t. the take-off position	The success ratio, the time, and the maximum deviation of the multirotor during the grabbing phase	**∼**	✓
	**Contact force control**	Apply a pushing force in horizontal direction against a wall with the end effector of the manipulator for at least 5 s	UAV position deviation	**∼**	✓
	**Position control**	Move the arm in the air to calculate the resultant position deviation of the UAV	The maximum position deviation, $\varepsilon_{UAV}$ *, and *t*_10%_†	**∼**	✓
	**Jammability**	Grab an object located at a given distance and height w.r.t. the take-off position, move the object to an specified height and hold it stably	Deviations in CoG of the AMS during (a) approaching and (b) manipulating phases	**∼**	✓
	**Energy Performance**	Grab an object located at a given distance and height w.r.t. the take-off position (in both confined and open spaces)	The average discharge of the battery (Ah)	✓	✓

* $||\varepsilon_{\mathrm{UAV}}||=||r_{\mathrm{UAV}}^{\mathrm{ref}}-r_{\mathrm{UAV}}$ , $r_{\mathrm{UAV}}||$ : position of UAV, $r_{\mathrm{UAV}}^{\mathrm{ref}}$ : reference position.

† $t_{10\%}$ is the elapsed time until $||\varepsilon_{\mathrm{UAV}}||\leq \;0.1L$ , L : reach of the manipulator.

Generally, the aerial robotic manipulators need to be as light and low inertia as possible because of severe dynamic coupling with the floating base. One of the main challenges of AMSs is the CoG variation of the aerial platform due to the attachment of the aerial manipulator and, more severely, by the dynamics of the manipulator in the air. In addition to their lower weight and inertia, continuum robots, because of their flexibility, have the advantage of taking the optimal form to have minimal effects on the CoG of the flying base. They can also jam, just underneath the aerial platform, when there is no need to manipulate. It will benefit the dynamics and stability of the flying platform, at least during hovering and approach.

Regarding agility, it is defined as the system’s ability to perform quick direction changes, as well as fast stops and starts (Bowling, 2006). In confined/unknown environments, the robot’s tasks involve navigating around obstacles by coordinating both quick translational and rotational motions, necessitating an agile manipulator. Besides, agile manipulators will spend less time performing the given task.

Energy performance is another feature that greatly contributes to the performance and functionality of AMSs. It directly depends on the energy consumption of the AMS. The lower the energy consumption is, the higher becomes the energy performance. Lower energy consumption means longer achievable mission time/distance. Two factors highly affect the energy performance: the AMS weight and its agility. Reducing the payload will increase the energy performance. Regarding the latter factor, higher agility leads to lower energy consumption over the mission as agile manipulators will spend less time performing the given task.

Regarding the above features and metrics, we can compare the ACMSs with conventional rigid-link manipulators (Table 1) as follows:

• Trajectory accuracy. We cannot compare robots’ structures based on this feature as it highly depends on other factors than the structure of the robot.
• Positioning accuracy and repeatability. More details and information is necessary. However, more control effort is usually needed to maintain the position accuracy and repeatability of CRs (Cowan and Walker, 2013).
• Payload capacity. Rigid-link manipulators have a larger payload capacity than continuum manipulators. However, CRs usually provide higher payload/weight ratio than rigid-link manipulators (Singh and Krishna, 2014).
• Force/load estimation and control. Rigid-link manipulators would benefit from producing higher force amplitudes. However, ease of shape-based force-estimation along with lower rise time and overshoot of CRs will provide considerable advantages over their rigid-link counterparts (Rucker and Webster, 2011).
• Collision detection and reaction. This advantage of CRs has been clearly demonstrated in the literature (Bajo and Simaan, 2011).
• Agility. In general, due to their links’ rigidity, conventional rigid-link robots provide superior agility, supporting rapid manipulation tasks.
• Object grasping. The shape adaptability of CRs will earn a better success rate (Zimmer et al., 2019). Furthermore, the built-in compliance of CRs suggests their enhanced performance, particularly robustness to the uncertainties/deviations of UAVs/objects position and object’s shape.
• Contact force control. Compliance of CRs will reduce the contact impact on position of the aerial platform, yielding better performance with CRs (Suarez et al., 2021).
• Multirotor position control. For the same reason as above, a better performance with CRs is expected. The built-in compliance will also reduce $t_{10}\%$ in CRs as well.
• Jammability. The CoG deviation of CRs could be much lower than rigid-link manipulators as they can easily pack and bring up the CoG of the manipulator just beneath the aerial platform. CRs could also optimize their form to adopt minimum overall CoG deviation during manipulation. Therefore, this will translate into lower negative impacts on dynamics and stability of AMSs.
• Energy performance: While continuum robots possess lower weights (and hence require smaller UAVs to carry them), rigid robots offer better agility (with less time spent for manipulation tasks), both impacting the consumed energy positively.

## Conclusion

In this article, we introduced the concept of ACMSs with variety of assemblies and applications. The focus was placed on the functionality of manipulation and the contributions of CRs into the context of aerial manipulation. Additionally, benchmarks for evaluating the performance of ACMSs were presented and the advantages of ACMSs over their conventional rigid-link counterparts were given. The introduced concept can considerably improve the aerial manipulation performance, particularly in the missions involving manipulation in confined and unstructured environments and/or interactions with humans.

The presented work has opened several challenging subjects that need to be addressed in future works. Continuum nature of arms present notable challenges in coupled kinematic/dynamic modeling of the whole system. Nonlinear and coupled nature of the system dynamics along with modeling uncertainties introduce significant difficulties into free and compliant motion control design for ACMSs. Additionally, robustness to disturbances such as wind gusts are more pronounced in control and planner designs for ACMSs which are equipped with flexible and light arms. Finally, the proposed platforms might face different challenges and constraints enforced through their implementation and will also require further studies pertaining to their usability in a variety of missions.

## Data Availability

The original contributions presented in the study are included in the article. Further inquiries can be directed to the corresponding author.
